# Half-Sandwich Arene Ruthenium(II) Thiosemicarbazone Complexes: Evaluation of Anticancer Effect on Primary and Metastatic Ovarian Cancer Cell Lines

**DOI:** 10.3389/fphar.2022.882756

**Published:** 2022-05-10

**Authors:** Seminay Guler, Hulya Ayar Kayali, Egemen Orkun Sadan, Betul Sen, Elif Subasi

**Affiliations:** ^1^ Izmir Biomedicine and Genome Center, Izmir, Turkey; ^2^ Izmir International Biomedicine and Genome Institute, Dokuz Eylul University, Izmir, Turkey; ^3^ Department of Chemistry, Division of Biochemistry, Faculty of Science, Dokuz Eylul University, İzmir, Turkey; ^4^ Institute of Science and Technology, Dokuz Eylul University, Izmir, Turkey; ^5^ Department of Physics, Faculty of Science, Dokuz Eylul University, Izmir, Turkey; ^6^ Department of Chemistry, Faculty of Science, Dokuz Eylul University, Izmir, Turkey

**Keywords:** organoruthenium(II)-arene complexes, thiosemicarbazone, antitumor activity, ovarian cancer cell lines, crystal structure

## Abstract

In this study, we describe the synthesis, characterization and antiproliferative activity of three organo-ruthenium(II) half-sandwich complexes [RuCl(η^6^-*p*-cym)(*N,S*-L)]Cl (I, II, and III). To form these complexes, three thiosemicarbazone ligands (TSCs) were synthesized; L = 5-nitro-2-carboxyaldehyde-thiophen-*N*-methyl-thiosemicarbazone, (L1); 2-acetyl-5-bromo-thiophen-*N*-methyl-thiosemicarbazone, (L2) and 2-acetyl-5-bromo-thiophen-*N,N*-dimethyl-thiosemicarbazone, (L3). The isolated compounds were analyzed using spectroscopic techniques such as elemental analysis, conductance measurements, FT-IR, ^1^H NMR spectroscopy, MALDI-TOF mass spectrometry, and single-crystal XRD. Our results demonstrated that the synthesized thiosemicarbazone ligands (TSCs) are bound to the metal ion as a bidentate ligand that coordinates through the thiocarbonyl sulfur and azomethine nitrogen atoms in all complexes (I, II, and III). The X-ray crystal structures of L1 and L2 revealed that both compounds are crystallized in the triclinic crystal system with space group P-1. The biological potency of newly synthesized TSC ligands (L1, L2, and L3) and their corresponding ruthenium complexes (I, II, and III) were investigated on human primary ovarian (A2780) and human metastatic ovarian (OVCAR-3) cell lines. To get detailed information respecting antitumor properties, cytotoxicity, DNA/BSA binding affinity, cellular uptake, DNA binding competition, and trans-epithelial resistance measurement assays were performed. Our results demonstrate that newly synthesized ruthenium(II) complexes possess potential biological activity. Moreover, we observe that the ruthenium complexes reported here show anticancer activity on primary (A2780) and metastatic (OVCAR-3) ovarian cancer cells.

## 1 Introduction

Cancer is a complex disease that is often difficult to diagnose and has the ability to spread to other organs easily, which significantly lowers the patient’s survival rate. Considered as one of the most deadly diseases in the world, it has been reported that one in three people develop cancer during their lifetime (Chakraborty and Rahman, 2012). Ovarian cancer has the highest mortality rate among the cancer types of the female reproductive system. According to the latest report of American Cancer Society Cancer Facts and Figure 2021, more than 21,000 women will be diagnosed with ovarian cancer in the United States and approximately 13,770 patients will die, ranking ovarian cancer fifth in cancer deaths among women. Due to the absence of early symptoms, nearly 80% of ovarian cancer cases are diagnosed at late stages, with a 5-years survival rate of less than 40%. Surgery still continues as the main standard treatment option for patients diagnosed with ovarian cancer (American Cancer Society, 2020). Since the introduction of chemotherapeutics as first-line cancer treatment after surgery, various metal-based therapeutics have been of great attention as novel antitumor drugs in the last decades (Zeng et al., 2017). However, there is increasing evidence showing that chemotherapeutics may have high toxic effects, cause drug resistance, and have side effects that decrease the patient’s quality of life (Barry and Sadler, 2013). Therefore, in order to overcome the drawbacks related to current chemotherapeutics, researchers have focused their attention on discovering new potential drugs that are less toxic, highly biocompatible, and highly selective for the tumors. In this context, ruthenium complexes have raised researchers’ interest over years, and they have been tested on a number of cancer cell lines. Ruthenium complexes possess interesting biological advantages such as low toxicity, potent efficacy, and lower occurance of drug resistance, and are considered as promising alternative candidates for the effective treatment of cancer (Liu et al., 2019). Up to now, many studies showed that ruthenium complexes have potent growth inhibitor activity on various cancer cells, including lung (Bugarcic et al., 2009), pancreas (Habtemariam et al., 2006), breast (Iida et al., 2016), and colon (Savic et al., 2020). Despite the synthesis of many ruthenium complexes that are effective in different types of cancer, only three of them (NKP1339, NAMI-A, and TLD1433) have shown efficient potency to reach to the clinical trials. NKP1339 and NAMI-A have been developed as chemotherapeutic agents, while TLD1433 has photosensitizer property for photodynamic therapy. Among these three, only NAMI-A has completed phase II clinical trials (Coverdale et al., 2019).

In the last decades, attention on Ru(II)-arene complexes has significantly increased in the cancer field. These complexes have versatile pharmacological features. The arene ligands can be used to increase the stability of Ru(II) complexes. These ligands can also be utilized to provide a lipophilic/hydrophilic balance to the compounds. Moreover, different co-ligands are known to influence the activity of Ru(II)-based compounds. Small variations in the structure of the ligands can greatly change the biological activities. As a result, various ligands have been considered to be used in the studies that use Ru(II)-arene complexes (Ashraf et al., 2016).

For the design of effective anticancer agents, the successful strategy would be design Ru(II)-arene complexes that include different variations of bioactive ligands. In this way, the creation of a synergistic effect between the ligand and the metal center may increase the biological activity of the complex. TSCs have been of interest due to their different biological properties (Su et al., 2018). Moreover, TSCs are in the class of *N*, *S* chelating ligands, therefore metals can be coordinated with TSC ligands. Ru(II) complexes that have TSC ligands have been proposed to have potent anticancer activity (Romero-Canelón et al., 2013; Su et al., 2015).

In the current study, we synthesized new TSC derivatives, [L = 5-nitro-2-carboxyaldehydethiophen-*N*-methyl-thiosemicarbazone, (L1); 2-acetyl-5-bromo-thiophen-*N*-methyl-thiosemicarbazone, (L2) and 2-acetyl-5-bromo-thiophen-*N,N*-dimethyl-thiosemicarbazone, (L3)] and their Ru (*p*-cym) complexes, [RuCl(η^6^-*p*-cym) (*N, S*-L)]Cl (I, II and III). Our results indicate that these newly synthesized Ru(II) complexes may have promising biological activity.

## 2 Materials and Methods

### 2.1 Materials

All commercial reagents were used without any treatment. RuCl_3_·nH_2_O, *N*-methyl/*N,N*-dimethyl-*3*-thiosemicarbazide, 2-acetyl-5-bromo-thiophen, 5-nitro-2-carboxyaldehydethiophen, α-phellandrene and other chemicals were purchased from Sigma-Aldrich. In all reactions, Schlenk techniques were used. Proper reaction conditions were maintained under an argon atmosphere. The dry solvents stored under inert gas were purchased from Sigma Aldrich. [{RuCl(η^6^-*p*-cym)}_2_ (μ-Cl)_2_] was synthesized following literature procedures (Bennett and Smith, 1974).

### 2.2 Methods

LECOCHNS-O-9320 and Varian 1000 FT spectrophotometer were used for the elemental and FT-IR analysis, respectively. HP Digital FT NMR (400 MHz) was used for ^1^H NMR data and reference was made to tetramethylsilane (TMS). 1,8,9-trihydroxyanthracene was used as a matrix for the mass analyses. Analyzes were recorded using a Bruker MALDI–TOF spectrometer. Conductance measurements were recorded on a Systronics automatic precision bridge 304 conductivity meter.

### 2.3 Synthesis of the Thiosemicarbazone Ligands

The synthesis of all ligands was carried out by following the given literature procedure (Yaman et al., 2017). The aldehyde (1-mol equivalent) chosen for the start of the reaction was dissolved in hot absolute ethanol solution (50 ml) and ambient conditions were created by a few drops of concentrated sulfuric acid. The same amount of the corresponding thiosemicarbazide (1 mol eq.) was added to the reaction medium. The reaction mixture was heated under reflux for 4 h. After the reaction was terminated, the synthesized TSC ligands were obtained as a precipitate after the solution was cooled to ambient temperature. Solids obtained after filtration was washed with ethanol several times and dried under vacuum.

#### 2.3.1 5-Nitro-2-Carboxyaldehydethiophen-*N*-Methyl-Thiosemicarbazone, L1

Orange powder, yield: 90%. C_7_H_8_N_4_S_2_O_2_: Calc. %C: 34.42; H: 3.30; N: 22.93; O: 13.10; S: 26.25. Found: %C: 34.82; H: 3.17; N: 22.66; O: 13.87; S: 26.48. FTIR (KBr pellet), ʋ/cm^−1^: 3339 (s), 3142 (s), 3105 (s) (ν(N (1)H + N(2)H)_sym_), 1564 (s), 1537 (s) ν(C = N), 1037 (s) ν(N-N), 908 (s) ν(C = S), 731 (s), 694 (s) (thiophen ring stretchings). ^1^H NMR (400 MHz, DMSO-*d*_6_) δ 11.81 (s, 1H, **H**C = N), 8.51 (s, 1H, N(2)**H**), 8.18 (s, 1H, N(1)**H**), 8.05 (d, *J* = 4.26, 1H, thiophen ring protons); 7.49 (d, *J* = 4.26, 1H, thiophen ring protons), 2.99 (d, *J* = 4.40, 3H, NH**CH**_**3**_). MALDI-TOF MS: m/z = 244 [M^+^]. Crystals were get by ethanol solution of L1.

#### 2.3.2 2-Acetyl-5-Bromo-Thiophen-N-Methyl-Thiosemicarbazone, L2

Yellow powder, yield: 85%. C_8_H_10_N_3_S_2_Br: Calc. %C: 32.88; H, 3.45; N, 14.38; S, 21.95. Found: %C: 32.23; H, 3.12; N, 14.22; S, 21.44. FTIR (KBr pellet), ʋ/cm^−1^: 3370 (s), 3175 (m), 3062 (m) (ν(N (1)H + N(2)H)_sym_), 1547 (s), 1496 (s) ν(C = N), 1045 (s) ν(N–N), 974 (s) ν(C = S), 798 (s) and 694 (s) (thiophen ring stretchings). ^1^H NMR (400 MHz, DMSO-*d*_6_) δ 10.30 (s, 1H, N (2)**H**), 8.06 (s, 1H, N (1)**H**), 7.29 (d, *J* = 4.00, 1H, thiophen ring proton); 7.18 (d, *J* = 4.00, 1H, thiophen ring proton), 3.01 (d, *J* = 4.40, 3H, NH**CH**_**3**_), 2.26 (s, 3H, N = CC**H**_3_). MALDI-TOF MS: m/z = 292 [M^+^]. Crystals were gained by slow evaporation of ethanol solution of L2.

#### 2.3.3 2-Acetyl-5-Bromo-Thiophen-N, N-Dimethyl-Thiosemicarbazone, L3

Light brown powder, yield: 84%. C_9_H_12_N_3_S_2_Br: Calc. %C: 38 78; H: 4, 07; N: 16,96; S: 20.91. Found %; C: 38, 22; H: 4, 86; N: 16, 75; S: 20.25. FTIR (KBr pellet), ʋ/cm^−1^: 3351 (s), 3236 (m), 3165 (m) (ν(N(2)H)_sym_), 1515 (s), 1488 (s) ν(C = N), 1064 (s) ν(N–N), 975 (s) ν(C = S), 789 (s), 705 (s) (thiophen ring stretchings). ^1^H NMR (400 MHz, DMSO-*d*_6_) δ 9.65 (s, 1H, N (2)**H**), 7.34 (d, *J* = 3.97, 1H, thiophen ring protons); 7.20 (d, *J* = 3.97, 1H, thiophen ring protons), 3.22 (s, 6H, N(**CH**_**3**_)_2_), 2.31 (s, 3H, N = CC**H**_3_). MALDI-TOF MS: m/z = 306 [M^+^].

### 2.4 Synthesis of the Complexes (General Procedure)

2-mol equivalents of TSC ligand were dissolved in dry methanol (20 ml). Then, 1 drop of HCl 37% was joined to the solution, and acidification of the reaction medium was achieved. In another schlenk [{RuCl(η^6^-*p*-cym)}_2_ (μ-Cl)_2_] (1-mol equivalent) was dissolved in 10 ml of dry DCM and the solution was added to the TSC ligand. The reaction was continued for 24 h under nitrogen with stirring at ambient temperature. After the reaction was terminated, the volume of solution was halved using a rotary evaporator. Diethyl ether was added until a solid precipitate was observed. The product was obtained by filtration and dried in vacuo.

[*RuCl*(*η* ^*6*^ *-p-cym*) (*N,S-L1*)]*Cl, I*.

Light brown powder, yield: 62%. RuC_17_H_22_N_4_O_2_S_2_Cl_2_: Calc. %C: 24.21; H, 3.05; N, 14.12; O, 8.06; S, 16.16. Found %C: 24.02; H, 3.56; N, 14.34; O, 8.72; S, 16.24. FTIR (KBr pellet), ʋ/cm^−1^: 3384 (m), 3099 (m), 3064 (m) (ν(N (1)H + N (2)H)_sym_), 1576 (s), 1544 (s), ν(C = N), 1033 (s) ν(N–N), 876 (s) ν(C = S), 733 (s), 637 (s) (thiophen ring stretchings). ^1^H NMR (400 MHz, DMSO-*d*_6_) δ 8.83 (s, 1H, **H**C = N), 8.11 (s, 1H, N(2)**H**), 7.62 (s, 1H, N(1)**H**), 7.12–7.05 (m, 2H, thiophen and 4H, *p*-cym ring protons), 3.07 (d, *J* = 4.26, 3H, NH**CH**_**3**_), 2.81-2.79 (m, 1H, *p*-cym C**H**Me_2_), 2.24 (s, 3H, *p*-cym C**H**_3_), 1.18 (d, *J* = 5.50 Hz, 6H, *p*-cym CH**Me**_2_). MALDI-TOF MS: m/z = 515 [M-Cl]^+^; Ʌ_M_ (Ω^−1^. m^2^. M^−1^) 75.

[*RuCl*(*η* ^*6*^ *-p-cym*) (*N,S-L2*)]*Cl,* ***II*.**

Light brown powder, yield: 53%. RuC_18_H_24_N_3_S_2_Cl_2_Br: Calc. %C: 24.30; H, 3.17; N, 9.45; S, 14.42. Found: %C: 24.19; H, 3.12; N, 9.14; S, 14.03. FTIR (KBr pellet), ʋ/cm^−1^: 3385 (w), 3162 (w), 3015 (m) (ν(N (1)H + N (2)H)_sym_), 1584 (s), 1564 (s) ν(C =N), 1034 (s) ν(N–N), 875 (s) ν(C = S), 789 (s), 736 (s) (thiophen ring stretchings). ^1^H NMR (400 MHz, DMSO-*d*_6_) δ 7.84 (s, 1H, N (2)**H**), 7.61 (s, 1H, N(1)**H**), 7.04–7.10 (m, 2H, thiophen and 4H, *p*-cym ring protons), 3.03 (d, *J* = 4.28, 3H, NH**CH**_**3**_), 2.87–2.79 (m, 1H, *p*-cym C**H**Me_2_), 2.48 (s, 3H, N=CC**H**_3_), 2.23 (s, 3H, *p*-cym C**H**_3_), 1.15 (d, *J* = 6.60 Hz, 6H, *p*-cym CH**Me**_2_). MALDI-TOF MS: m/z = 547 [M-Cl-Me]^+^; Ʌ_M_ (Ω^−1^. m^2^. M^−1^) 72.

[*RuCl(η* ^*6*^ *-p-cym*) *(N,S-L3*)]*Cl,* ***III*.**

Orange powder, yield: 66%. RuC_19_H_26_N_3_S_2_Cl_2_Br: Calc. %C: 26.18; H, 3.51; N, 9.16; S, 13.98. Found %C: 26.32; H, 3.22; N, 9.03; S, 13.25. FTIR (KBr pellet), ʋ/cm^−1^: 3387 (m), 3043 (m), 2957 (s) (νN (2)H)_sym_), 1572 (s), 1535 (s) ν(C=N), 1032 (s) ν(N-N), 900 (s) ν(C = S), 800 (s), 674 (s) (thiophen ring stretchings). ^1^H NMR (400 MHz, DMSO-*d*_6_) δ 7.77 (s, 1H, N (2)**H**), 7.05–7.10 (m, 2H, thiophen and 4H, *p*-cym ring protons), 2.77–2.80 (m, 1H, *p*-cym C**H**Me_2_), 2.48 (s, 3H, N = CC**H**_3_), 2.23 (s, 3H, *p*-cym C**H**_3_), 2.07 (s, 6H, N(**CH**_**3**_)_2_), 1.16 (d, *J* = 5.2 Hz, 6H, *p*-cym CH**Me**_2_). MALDI-TOF MS: m/z = 562 [M-Cl-Me]^+^; Ʌ_M_ (Ω^−1^. m^2^. M^−1^) 78.

### 2.5 Determination and Refinement of the Crystal Structure

The molecular and crystal structures of L1 and L2 were elucidated by single-crystal X-ray diffraction method. Rigaku-Oxford Xcalibur diffractometer with an Eos CCD area detector at 150 K has been used to collect the single-crystal data of both compounds. The measurements were performed by an ω-scan technique using graphite–monochromated MoK_α_ radiation (λ = 0.71073 Å) from an enhanced X-ray source. CrysAlis^Pro^ program (Agilent and CrysAlis PRO, 2014) has been utilized to collect and reduce the data, as well as to handle the cell refinement. For the solution of the crystal structure and to determine the space group, we have used the ShelXT (Dolomanov et al., 2009) structure solution program with Intrinsic Phasing. The full-matrix least-squares method based on *F* ^*2*^ against all reflections by using the SHELXL (Sheldrick, 2015) has been employed to refine the coordinates and anisotropic thermal parameters of non-hydrogen atoms. These calculations are carried out under the crystal structure crystallographic software package OLEX2 system (Dolomanov et al., 2009). Anisotropic thermal parameters were applied to all non-hydrogen atoms. PLATON software was used to calculate and to analyze the geometrical results (Spek, 2003). The pictures have been created by OLEX2 tools. (Dolomanov et al., 2009). The crystal structure of L1 was determined as a two-component non-merohedral twin with the final ratio of the twin domains being 0.7790(6):0.2210(6). The non-merohedral twinning was taken into account during the data reduction and structure refinement using the data in HKLF5. The final BASF parameter describing the ratio of the two twin components is 0.221. The R_int_ value is not available in the Table 1 due to merging twin components with MERGE 0. The concise crystal data, data collection, and structure refinement for both compounds L1 and L2 are displayed in Table 1.

**TABLE 1:** Crystal data and structure refinement parameters for complexes L1 and L2.

	L1	L2
Chemical formula	C7H8N4O2S2	C8H10BrN3S2
Formula weight	244.29	292.22
Temperature (K)	150.01 (10)	150.01 (10)
Space group	P-1	P-1
Crystal system	Triclinic	Triclinic
a (Å)	4.4615 (6)	7.1576 (5)
B (Å)	9.4095 (13)	7.6088 (7)
c (Å)	13.137 (2)	11.7561 (11)
α (^o^)	72.024 (14)	93.901 (8)
β (^o^)	82.613 (13)	99.191 (7)
γ (^o^)	77.432 (12)	116.951 (8)
Cell volume (Å^3^)	510.89 (13)	556.16 (8)
Formula unit cell Z	2	2
ρ_calc_ (g**/**cm^3^)	1.588	1.745
F (000)	252.0	292.0
μ (mm^−1^)	0.506	4.035
Crystal size (mm^3^)	0.484 × 0.089 × 0.039	0.506 × 0.386 × 0.225
Diffractometer	Xcalibur, Eos	Xcalibur, Eos
Radiation**/**Wavelength (Å)	MoKα/0.71070	MoKα/0.71070
Reflections measured	2669	2988
Range of *h, k, l*	−5 ≤ h ≤ 5	−8 ≤ h ≤ 8
	−11 ≤ k ≤ 11	−8 ≤ k ≤ 9
	−16 ≤ l ≤ 15	−14 ≤ l ≤ 10
Independent reflections	2669 [Rint = N/A	2103 [Rint = 0.0232
	Rsigma = 0.1238]	Rsigma = 0.0538]
Data**/**restraints**/**parameters	2669/0/138	2103/0/129
Final R indexes [I ≥ 2σ (I)]	R1 = 0.0456, wR2 = 0.0589	R1 = 0.0357, wR2 = 0.0727
Final R indexes [all data]	R1 = 0.0809, wR2 = 0.0633	R1 = 0.0443, wR2 = 0.0764
Goodness-of-fit on F^2^	0.823	1.027
Largest diff. Peak**/**hole (e Å^−3^)	0.32/−0.29	0.55/−0.51

### 2.6 Cell Culture

The human primary ovarian cancer cell line (A2780) and the human metastatic ovarian cancer cell line (OVCAR-3) were obtained from the American Type Culture Collection (ATCC). Cells were grown in RPMI 1640 medium supplemented with 10% fetal bovine serum (FBS), penicillin (100 units/mL), and streptomycin (100 µg/ml). The cells were cultivated at 37°C in a humidified 5% CO_2_ incubator.

### 2.7 *In vitro* Cytotoxicity Assay

The antiproliferative effect of the newly synthesized TSC ligands, Ru(II) complexes, and chemotherapeutic drugs (carboplatin, oxaliplatin, paclitaxel) as references were determined by the 3-(4,5-dimethylthiazol-yl)-2,5-diphenyltetrazolium bromide (MTT) assay. Stock solutions of TSC ligands and Ru(II) complexes were freshly prepared in dimethyl sulfoxide (DMSO) at 5 mM concentration. Briefly, cells reaching approximately 80% confluency were placed into 96-well plates (7,500 cells/100 µl/well) and waited for nearly 16–18 h. Varying concentrations of TSC ligands (25, 50, and 100 µM) and Ru(II) complexes (1, 5, 10, and 50 µM) were applied to the cells for 24 h. Then, the culture medium was taken and MTT (5 mg/ml) solution was put (100 μL/well) to each well. The plates were incubated for 4 h at 37°C. Then, the MTT solution in each well was carefully discarded and Dimethyl sulfoxide (DMSO) was placed in the wells. The absorbance was obtained by using a microplate reader (Thermo Multskan Go) at 540 nm. The experiments were performed in three biological replicas. IC_50_ values were defined as the drug concentration which limits cell growth at a 50% ratio and IC_50_ values were calculated by using cell survival diagrams.

### 2.8 Cellular Uptake

Effective uptake of Ru(II) complexes into the cell were evaluated by fluorescence microscopy. Briefly, cells reaching approximately 70% confluency were counted with trypan blue and placed in 6-well plates with sterile coverslips containing 5 × 10^4^ cells in each well, then cells were kept in an incubator for overnight to adhere to the surface. Ru(II) complexes were applied to the cells at a certain concentration (20 µM) for 24 h. Cells were then washed with 1X phosphate salt buffer (PBS) and permeabilized with 4% paraformaldehyde (PFA) that contains 0.1% Triton-X-100. The cells were kept in an incubator at 25°C for 15 min. After incubation, the cells with increased pore permeability were applied in a 1: 2000 ratio of 4 6, 6-diamidino-2- phenylindole dihydrochloride (DAPI) (5 mg/ml) and were kept in the dark for 5 min at room temperature. After washing again with 1X PBS, coverslips were carefully covered on slides, and images were taken with a fluorescence microscope.

### 2.9 DNA Binding Assay

DNA binding assay was performed in 1 cm quartz cuvettes. The temperature was set to 25°C. The absorption data were obtained using a UV-Vis spectrophotometer (UV-1800, Shimadzu). Calf Thymus DNA (CT-DNA) concentration was acquired at 260 nm. CT-DNA purity was measured at 260/280 nm. Experiments were performed in 5 mM Tris-HCl buffer including 50 mM NaCl (pH 7.2). 10 µM concentration of TSC ligands and Ru(II) complexes were standardized with 0.07–1.4 mM CT-DNA concentrations at 37°C for 1 h, and the spectrum was recorded between 200–800 nm.

### 2.10 Protein Binding Assay

The spectra of (BSA) in the presence and absence of ruthenium (II) complexes were carried out using the Spectrofluorophotometer (RF-5301 PC, Shimadzu) with excitation at 260 nm. The binding assay was performed in 50 mM phosphate buffer (pH 7.2). BSA emission changes were recorded between 250 and 450 nm by titrating the constant concentration of BSA (1 µM) with increasing Ru complex concentrations (0–50 µM).

### 2.11 DNA Binding Competition Experiments

To determine the binding modes of Ru(II) complexes on DNA, two different competitive binding studies were performed using DAPI and methyl green. In the DAPI competitive binding study, Ru(II) complexes (100 µM) diluted in 10 mM pH 7.4 phosphate buffer were incubated with CT-DNA (20 µM) and DAPI (15 µM- 5 mg/ml) at 23°C for 15 min. The samples were scanned at the excitation wavelength of 338 nm and the emission wavelength of 461 nm in the spectrophotometer (Thermo Varioskan). In the methyl green competitive binding study, 2% methyl green, CT-DNA (50 µM), and Ru(II) complexes (100 µM) dissolved in 7.5 mM MgSO4 containing 50 mM pH 7.5 Tris-HCl solution were kept in a water bath at 37°C for 24 h. The samples were scanned on the spectrophotometer at an excitation wavelength of 630 nm and emission wavelength of 677 nm. The absorption plot was drawn by comparing the absorbances in solutions containing Ru(II) complexes compared to the control group without Ru(II) complexes.

### 2.12 Trans-Epithelial Resistance Measurement

*Trans*-Epithelial Resistance of newly synthesized Ru(II) complexes was determined by using the *Trans*-epithelial resistance (TED) measurement device (Millicell®ERS-2, Millipore). Briefly, the cells were placed in transwells with a diameter of 0.4 µm pore at a concentration of 7,500 cells/250 µl medium. Approximately 1000 µl of growth medium was placed outside the transwells. The growth medium of the cells was changed every 3 days. Transepithelial resistance was measured before changing the medium. When the cells reached sufficient confluency after about 14 days, Ru(II) complexes were applied at IC_50_ concentrations, and differences in trans-epithelial resistance were observed by taking measurements at 24, 48, 72, 96, and 120 h.

## 3 Results and Discussion

### 3.1 Synthesis and Characterization of the Complexes

New ligands L = 5-nitro-2-carboxyaldehydethiophen-*N*-methyl-thiosemicarbazone, (L1); 2-acetyl-5-bromo-thiophen-*N*-methyl-thiosemicarbazone, (L2) and 2-acetyl-5-bromo-thiophen-*N, N*-dimethyl-thiosemicarbazone, (L3) were synthesized according to described procedures (Yildirim et al., 2014; Öztürk et al., 2014; Dömötör et al., 2018; Tavsan et al., 2018; Subasi et al., 2020). Then half-sandwich arene Ru(II) complexes (I−III) of the general formula Ru [(η^6^-*p*-cym) (*N, S*-L)Cl]Cl were isolated in good yields.

**SCHEME 1:**
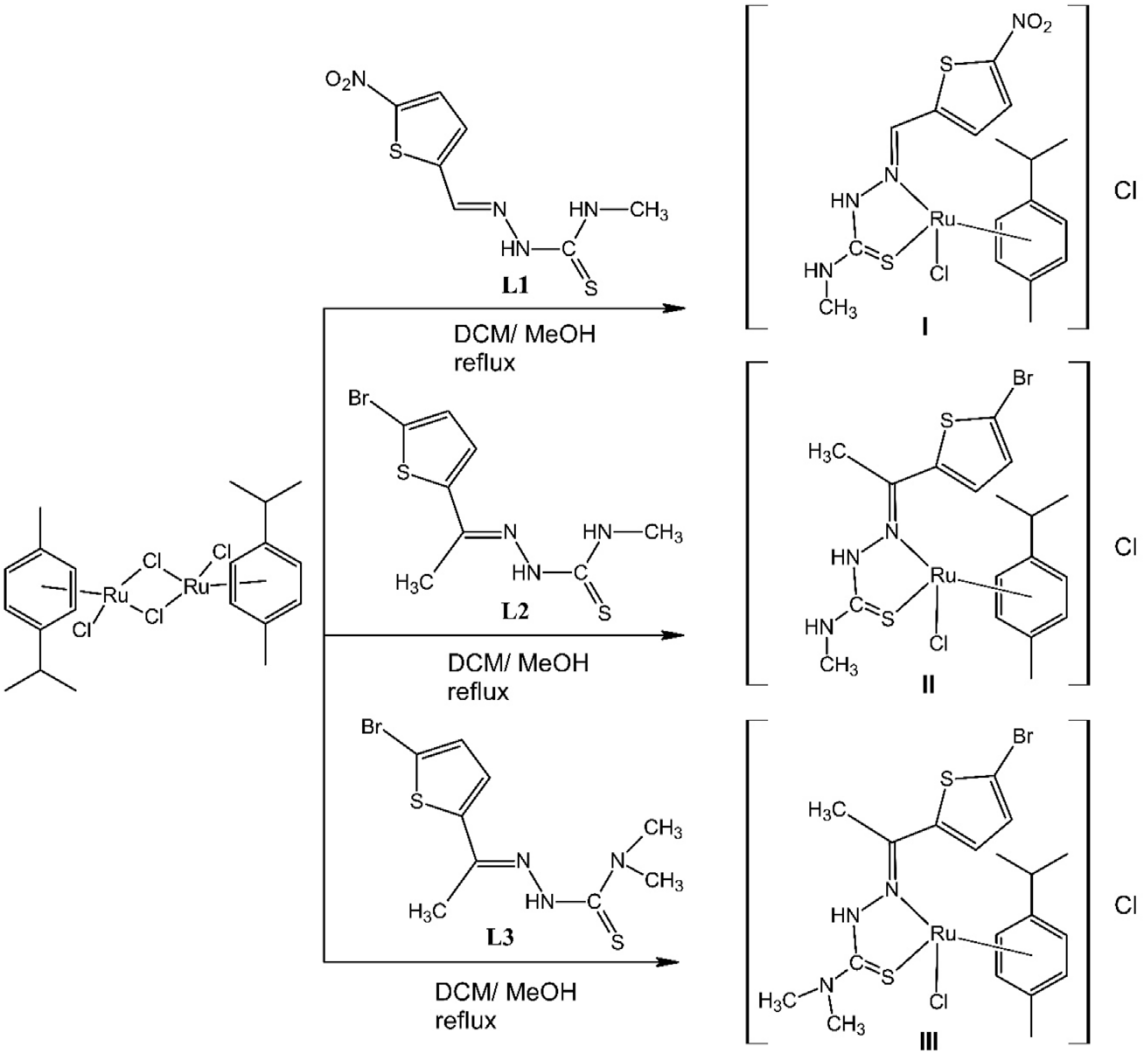
TSC ligands (L1, L2, and L3) and their ruthenium complexes (I, II, and III).

The identification of the complexes was confirmed by elemental analysis, FT-IR, ^1^H NMR spectroscopy, MALDI-TOF spectrometry, and L1 and L2 structures were confirmed by single-crystal X-ray crystallography. In all cases, the metal binds to a chloride ion, a η^6^-*p*-cym ring, and a N, S bidentate TSC chelating ligand (Scheme 1).

The infrared spectra were recorded in the range of 4,000–650 cm^−1^. TSCs which are shown with the general formula, [R(1)R(2)C(2) = N(3)N(2)(H)C(1) = (S)N(1)R(3)R(4)] commonly coordinate to metal as either of two tautomeric forms, a neutral thione form or the anionic thiol form. The existence of a band corresponds to the hydrazine N(2)–H group, which proposed the coordination of a TSC to the metal center in a neutral thione form, not an anionic thiol form (Subasi et al., 2020). The *ν*(C = N) stretching frequencies of the complexes (I, II, and III) showed changes compared with free ligands L1, L2, and L3 which support complexation formation. The absorptions due to azomethine C=N of the free ligands at 1564, 1537 cm^−1^ (L1); 1547, 1496 cm^−1^ (L2), and 1515, 1488 cm^−1^ (L3) were observed. Coordination of the TSCs to Ru(II) through imine nitrogen is expected to change the electron density in the azomethine and thus alter *ν*(C = N) band frequency (Manimaran and Jayabalakrishnan, 2012). *ν*(C= N) absorption frequencies were seen at 1576 and 1544 cm^−1^ (I); 1584 and 1564 cm^−1^ (II) and 1572 and 1535 cm^−1^ (III) in the FT-IR spectra of the complexes. The FTIR spectra of L1, L2, and L3 exhibited characteristic strong absorption bands attributed to thiocarbonyl (C = S) stretching, at 908, 974, and 975 cm^−1^ consecutively. They all were shifted to lower frequencies 876, 875, and 900 cm^−1^ for the complexes (I, II, and III). These shifts proved that the ligand coordinated as a neutral, bidentate through thiocarbonyl sulfur and imine nitrogen atoms in (I, II, and III). The *ν* (N-N) bands of TSCs were seen at 1037 cm^−1^, (L1); 1045 cm^−1^, (L2), and 1064 cm^−1^, (L3). The decrease in the frequency of these bands 1033 cm^−1^ (I), 1034 cm^−1^ (II), and 1032 cm^−1^ (III) confirms the coordination *via* the imine nitrogen as shown in the literature (Manivannan et al., 2007).

The L-to-M bonding is further supported by ^1^H NMR spectra. Two sets of signals of the *p*-cymene ring and the TSC protons of the coordinated ligand are seen definitely in the ^1^H NMR spectra. In the spectra of (I, II, and III) all indications were that the TSCs remained in the neutral form due to the presence of the N(2)H protons. The NH absorptions in the spectra of the complexes were essentially altered from that of the free ligands. Absorptions of the free ligands N(2)H protons were observed at 8.51, (L1); 10.30, (L2), and 9.65, (L3) ppm whereas upon coordination the signals appeared at 8.11, (I); 7.84, (II), and 7.77, (III) ppm with slight highfield shifts. An upfield shift compared to the free TSCs upon coordination to Ru(II) ion, confirming coordination of the metal ion to the imine nitrogen atom as the signal became more shielded in each case. This was also observed for similar arene Ru(II) TSC complexes (Raja et al., 2010; Stringer et al., 2011). In the spectrum of complex I, a sharp singlet at 8.83 ppm was assigned to azomethine proton (HC = N). The position of the azomethine signal in the complex was shifted to a higher field compared to free ligands at 11.81 ppm (L1), revealing coordination through the imine nitrogen. The absence of the thiol proton signal at 4.00 ppm is consistent with the idea that TSCs existed as the thione tautomer in the complexes (I, II, and III) (Das et al., 2010). The singlets due to the methyl moiety (CH_3_C = N) in L2 and L3 spectra were observed at around 2.26 and 2.31 ppm respectively, these absorptions moved slightly towards the upper field at 2.48 ppm for both of the complexes II and III.

The N(1)H singlet signals in the complexes, 7.62 ppm, (I) and 7.61 ppm, (II) attributed to the TSC were slightly changed to the higher field from the free ligands, 8.18 ppm, (L1) and 8.06 ppm, (L2). The N(1) (Me)H group generated doublets at 2.99 ppm, (L1) and 3.01 ppm, (L2) with a slight change in complexes at 3.07 ppm (I) and 3.03 ppm (II). Singlet resonances of NMe_2_ protons in L3 are at 3.22 ppm were present at 2.07 ppm in the NMR spectrum of (III) with gradual upfield shifts.

The *p*-cym protons resonated at frequencies typically observed for this group (Beckford et al., 2009). Multiplets belong to the *p*-cym, and thiophene ring protons were seen at around 7.12–7.05 ppm, (I); 7.04–7.10 ppm, (II); 7.05–7.10 ppm, (III) for the complexes. The isopropyl methines of *p-*cym ring were emerged as multiplets at 2.79–2.81 ppm, in (I); 2,87–2.79 ppm, in (II), and 2.77–2.80 ppm, in (III), consecutively. The singlet signals owing to the methyl group on the *p-*cym ring at 2.24 ppm (I), 2.23 ppm (II), and 2.23 ppm (III) in sequence. The isopropyl methyls appeared as doublets at 1.18 ppm (I), 1.15 ppm (II), and 1.16 ppm (III).

All the protons resonated in commonly expected regions. The ^1^H NMR spectra of (I, II, and III) are actually a direct combination of the signals from the ligands plus those from the p-cym moiety. In the ^1^H NMR spectra of (I, II, and III) all indications are that the ligands remain neutral form.

The MALDI-TOF mass spectra of the TSCs and the complexes proved the suggested structures. Exact molecular ion peaks were observed for the ligands at 244 m/z (L1), 292 m/z (L2), and 306 m/z (L3). However, according to the mass analysis of the complexes the peaks at 515 m/z (I), 547 m/z (II), and 562 m/z (III) were equal to the total molecular weight of [M-Cl]^+^, (I); [M-Cl-Me]^+^, (II); [M-Cl-Me]^+^, (III). The chloride ion loss confirmed all the complexes have chloride ions as a counter ion.

The complexes I-III have molar conductance (10^–3^ M in DMSO) in the range of 72–78 Ʌ_M_ (Ω^−1^. m^2^. M^−1^) at 38°C suggesting 1:1 electrolytic behavior (Ali et al., 2013).

### 3.2 Structural Description of Compounds L1 and L2

**FIGURE 1:**
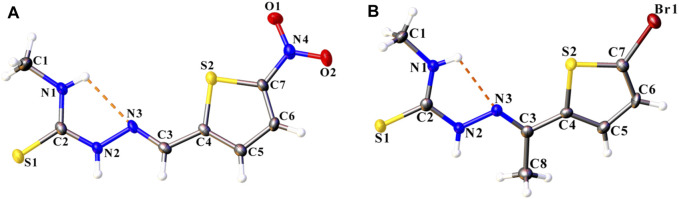
The molecular structure of compounds **(A)** L1 and **(B)** L2, with displacement ellipsoids drawn at the 50% probability level. The intramolecular N–H···N hydrogen bond is shown as orange dashed lines. H atoms are represented as small spheres of arbitrary radii.

Both ligand compounds L1 and L2 crystallized in the triclinic crystal system with space group P-1, and their molecular structures are depicted in Figures 1A,B. There are small differences between both compounds in the bond lengths and angles as shown in Table 2.

**TABLE 2:** Selected bond lengths (Å) and angles (o) for the compounds L1 and L2.

	L1	L2
S2–C7	1.713 (3)	1.720 (4)
S2–C4	1.726 (3)	1.740 (3)
S1–C2	1.686 (3)	1.682 (4)
N1–C2	1.312 (3)	1.321 (4)
N2–C2	1.360 (3)	1.358 (4)
N3–N2	1.366 (3)	1.381 (4)
C5–C6	1.405 (4)	1.407 (5)
N1–C2–N2	117.2 (3)	116.9 (3)
C3–C4–S2	121.8 (2)	120.4 (3)
S2–C7–N4/Br1	119.7 (2)	120.3 (2)
C4–S2–C7	89.2 (1)	90.8 (2)
C3–C4–C5	125.6 (3)	129.6 (3)

It is well known that thiosemicarbazones can show thione–thiol tautomerism due to the presence of the thioamide functional group (Nehar et al., 2016; Zhang et al., 2009). S–C and C–N bond lengths have supported that both compounds appear in as a thione form. The exocyclic S–C bond distances are 1.686 (3) Å in compound L1 and 1.682 (4) Å in compound L2, and can be compared with those corresponding to some other related complexes (Parsons et al., 2000; Latheef et al., 2006; Trinajstić, 1968). These bond lengths lie between the values of isolated C–S single and C = S double bond (1.82 and 1.56 Å, respectively) (Trinajstić, 1968), showing a partial double bond character (Miroslaw et al., 2011; Kumbhar et al., 1997) due to electron delocalization. On the other hand, C2–N2 bond distances are more or less the same for both compounds, and these distances are of single-bond character (Table 2). Similar values for C–N bond lengths are also observed for related compounds in the literature (Trinajstić, 1968; McBurney et al., 2005; Latheef et al., 2006).

Both compounds LI and L2 are is close to planar with the greatest deviation from the mean plane being 0.133 (3) at O1 and−0.236 (3) Å at C1, respectively. However, the 5-nitro thiophene ring in L1 [5-bromo thiophene ring in L2] and thiosemicarbazone fragment of the molecule were twisted concerning for to each other making a dihedral angle of 4.24 (6)° [9.085 (1)°].

N4–O1 and N4-O2 bond lengths are 1.221 (3) and 1.236 (3) Å in compound L1, which are in agreement with bond distances reported for other compounds of 5-nitrothiophene (Ceylan et al., 2011; Köysal et al., 2016; Pappenfus et al., 2018). The Br1–C7 bond length of 1.874 (3) Å is normal and similar to those reported in the literature (Bernstein et al., 1995; Li and Li, 2009; Geiger et al., 2014).

In the crystal packing of compounds L1 and L2, intermolecular interactions construct centrosymmetric dimeric motifs but give different supramolecular architecture. An S (5) ring motif is generated due to cyclic intramolecular N1–H1···N3 hydrogen bonds which support the molecular conformation of both compounds (Halfpenny and Sloman, 2000).

**FIGURE 2:**
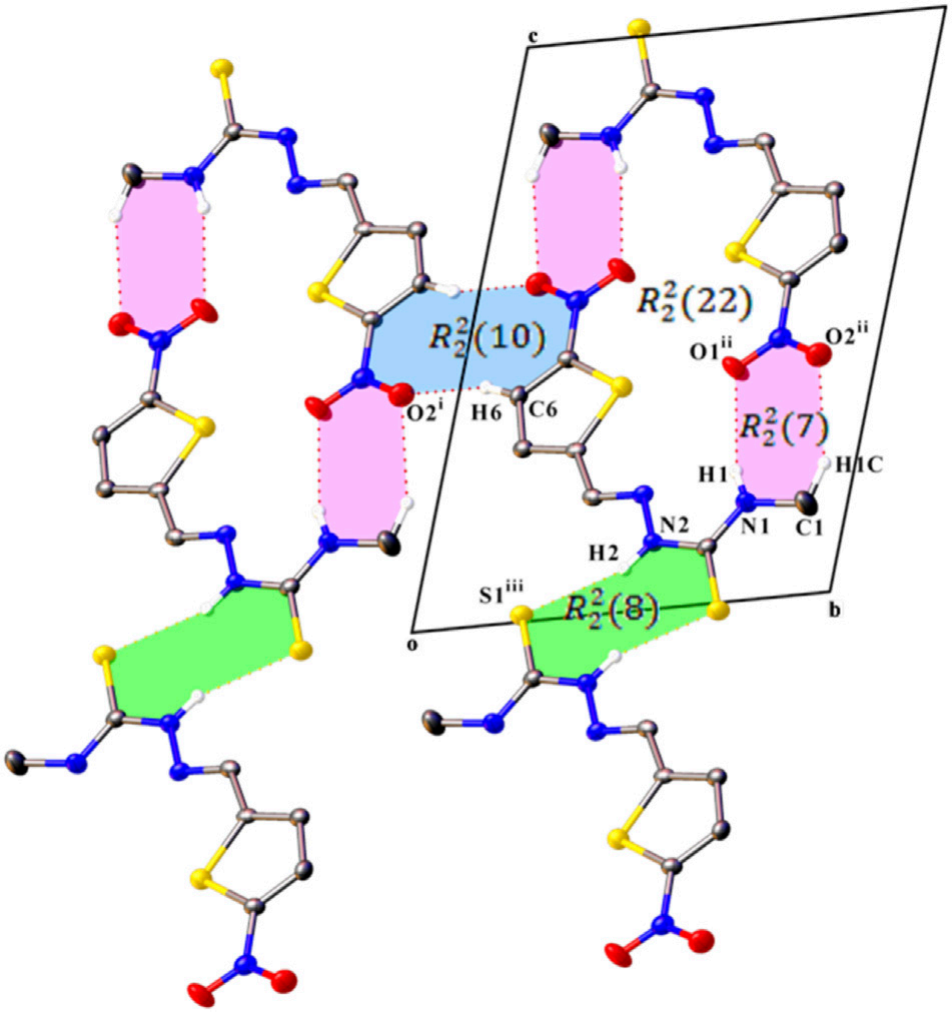
A partial view along the *a* axis of the crystal packing of compound L1, showing the formation of a cyclic R ring motifs formed by C–H⋯O, N–H⋯O, and N–H⋯S hydrogen bonds. H atoms not involved in hydrogen bonding have been omitted for clarity. Symmetry codes are as in Table 3.

In the crystal structure of L1, C6 atom act as hydrogen bond donor to O2 atom of an inversion-related molecule, producing an $R_{2}^{2}\left(10\right)$ hydrogen-bonded dimer through C6–H6∙∙∙O2 hydrogen bond. Additionally, a pair of N1–H1∙∙∙O1 and C1–H1C∙∙∙O2 interactions constitutes a cyclic dimer with an $R_{2}^{2}\left(7\right)$ loop while N1–H1∙∙∙O1 hydrogen bond also generates inversion dimer enclosing $\;R_{2}^{2}\left(22\right)$ rings. This dimer linkage is connected by another dimer-set which is linked via a pair of N2–H2∙∙∙S1 hydrogen bonds, a forming centrosymmetric dimer with an $R_{2}^{2}\left(8\right)$ ring motif. The molecules are formed a two dimensional hydrogen bonded network extending parallel to $\left(1\bar{1}2\right)$ through these intermolecular interactions (Figure 2). Furthermore, these aforementioned dimers are stacked by intermolecular N4–O2∙∙∙Cg (Cg is the centroid of thiophene ring) interactions along the diagonal of (110) plane, giving an overall three-dimensional structure.

**FIGURE 3:**
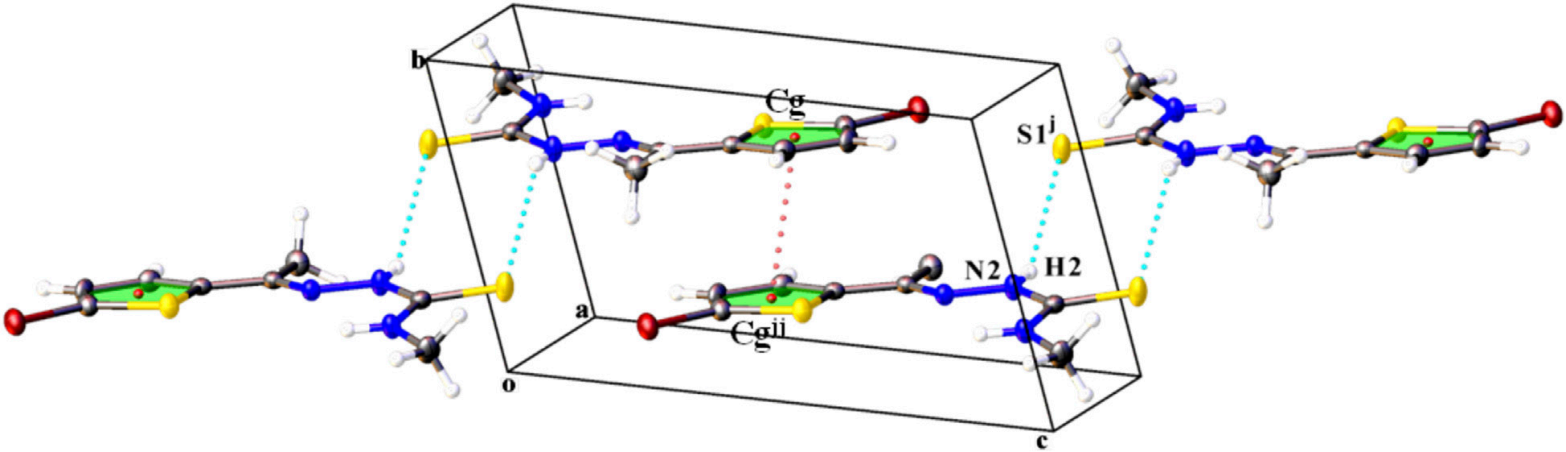
Part of the crystal structure of compound L2 showing the formation of hydrogen-. bonded dimers running parallel to the (110) plane, and stacking of layers linked by π ⋯π (thiophene ring) intermolecular interactions along the *b* axis. H atoms not involved in hydrogen bonding have been omitted for clarity. Symmetry codes: (***j***) 2−x,1−y,2−z; (***jj***) 1−x, 1−y, 1−z.

In the crystal of compound L2, N2–H2∙∙∙S2 hydrogen bond pairs give inversion dimer with a $R_{2}^{2}\left(8\right)$ ring motif, leading to stairs aligned parallel to the (110)  plane. The dimers are arranged in layers and are stacked into the crystallographic *b*-axis direction by π‒π interaction [Cg∙∙∙Cg^***jj***^ = 3.916 (2) Å, inter-planar distance = 3.529 (1) Å, slippage 1.697 Å, where Cg is the center of gravity of thiophene ring; symmetry code: (***jj***) 1−x,1−y,1−z] (Figure 3). In addition, Br1∙∙∙S1 inter-ligand distance is 3.4577 (3) Å which is much shorter than the corresponding expected van der Waals radii sum of 3.70 Å (Koziol et al., 1988). Hence, these distances may denote the presence of nonbonding intermolecular interactions. These interactions align the molecules along the diagonal line of the *b* and *c* axes. For both compounds, detailed information on the intra- and intermolecular interactions is given in Table 3.

**TABLE 3:** Hydrogen-bond geometry for compounds L1 and L2 (Å, o).

D—H ∙∙∙ A	D—H	H ∙∙∙ A	D ∙∙∙ A	D—H ∙∙∙ A
Compound L1				
N1–H1∙∙∙N3	0.86	2.29	2.657 (3)	106
C6–H6∙∙∙O2i	0.93	2.43	3.336 (3)	164
N1–H1∙∙∙O1ii	0.86	2.38	3.195 (3)	158
C1–H1C∙∙∙O2ii	0.96	2.47	3.340 (4)	150
N2–H2∙∙∙S1iii	0.86	2.54	3.362 (2)	159
N4–O2∙∙∙Cgiv	1.236	3.656	4.010 (3)	97.5
Compound L2				
N1–H1∙∙∙N3	0.86	2.22	2.608 (5)	107
N2–H2∙∙∙S1j	0.86	2.74	3.423 (3)	137

Symmetry transformations used to generate equivalent atoms: (i)−x,-y,1-z; (ii) 1−x,1−y,1−z; (iii) 3−x,1−y,-z; (iv)−1 +x,y,z; (j) 2−x,1−y,2−z.

### 3.3 Antiproliferative Activity

The MTT assay procedure was applied to evaluate the *in vitro* cytotoxicity of Ru(II) complexes on human A2780 (primary) and human OVCAR-3 (metastatic) ovarian cancer cell lines. Carboplatin, Oxaliplatin, and Paclitaxel were used as comparative reference substances, and were investigated under identical conditions. Both cell lines were treated with different concentrations of TSC ligands (25, 50, 100 µM) and Ru(II) complexes (1, 5, 10, 50 µM) for 24 h. The IC_50_ rate for Ru(II) complexes and reference substances against A2780 and OVCAR-3 cell lines were shown in Table 4.

**TABLE 4:** IC_50_ values of newly synthesized TSC ligands and Ru complexes at micromolar (µM) concentration (ND *:Non Detectable).

IC50 Concentration(µM)			
Compound	A2780	OVCAR-3	
Ligands	L1	ND*	ND*
	L2	0,8 ± 0,07	11,6 ± 0,2
	L3	0,3 ± 0,06	27,7 ± 0,4
Ru-complexes	I	2,1 ± 0,3	5,5 ± 0,4
	II	1,7 ± 0,3	3,7 ± 0,3
	III	2,1 ± 0,3	1,1 ± 0,2
Commonly used anticancer durgs	Paclitaxes	3,1 ± 0.09	46 ± 0,2
	Oxliplatin	0,9 ± 0.006	828,4 ± 0,1
	Carboplatin	48,7 ± 0,2	42 ± 0,2

Based on IC_50_ values, it was noticed that the highest cytotoxic activity on A2780 tumor cells was shown by complex II, and the highest cytotoxic activity on OVCAR-3 tumor cells was shown by complex III. This study showed that Ru(II) complexes (I, II, and III) were more cytotoxic on the OVCAR-3 cell line than chemotherapeutic agents used as reference substances (Carboplatin, Oxaliplatin, and Paclitaxel) in the recent study. The situation was slightly different considering the cytotoxicity experiments on the A2780 cell line. I and III complexes were more cytotoxic on the A2780 cell line than Paclitaxel and Carboplatin. However, Oxaliplatin showed the highest cytotoxic effect on A2780 cells than all Ru(II) complexes. When the IC_50_ values of the newly synthesized ruthenium (II) complexes obtained from the cytotoxicity experiments on both cell lines were examined, it was observed that the IC_50_ values of the Ru complexes on the A2780 cell line were lower. This showed that ruthenium complexes were more active in the primary ovarian cancer cell line. MTT results on ovarian cancer cell lines of ruthenium (II) complexes are in accordance with the literature studies (Tsovaltzi et al., 2017). Therefore this study suggested that newly synthesized organo ruthenium (II) complexes have a strong cytotoxic effect on primary (A2780) and metastatic (OVCAR-3) ovarian cancer cell lines.

### 3.4 DNA Binding Results

The development of new anticancer drugs requires the recognition of the DNA binding activities of the molecules. There are three different binding modes to DNA; major and minor groove binding, electrostatic or allosteric binding, and intercalation. The binding activity of the molecules has great importance for the *in vivo* potency (Kelly et al., 1985). In this study, the constant concentration (10 µM) of TSC ligands and Ru(II) complexes were titrated with different nucleic acid concentrations (0.07–1.4 mM). The reactions were performed in 5 mM Tris-HCl buffer including 50 mM NaCl (pH 7.2) buffer. The UV absorption rate at 260 and 280 nm for CT-DNA solutions was found to be 1.8–1.9, indicating no protein in DNA. It is known that Ru(II) complexes can interact with DNA containing aromatic ligands with 104–106 affinity (Li et al., 2014). Quantitative evaluation of DNA binding affinities was calculated using the equation [DNA]/(ɛa−ɛf) = ([DNA]/(ɛb − ɛf)+1/(Kb(ɛb–ɛf)) (Wolfe et al., 1987). Here [DNA] shows the DNA concentration, the adsorption coefficient according to, εf is the damping coefficient of the free form complex/ligands, εb is the damping coefficient of DNA-bound Ru(II) complexes/TSC ligands.

**FIGURE 4:**
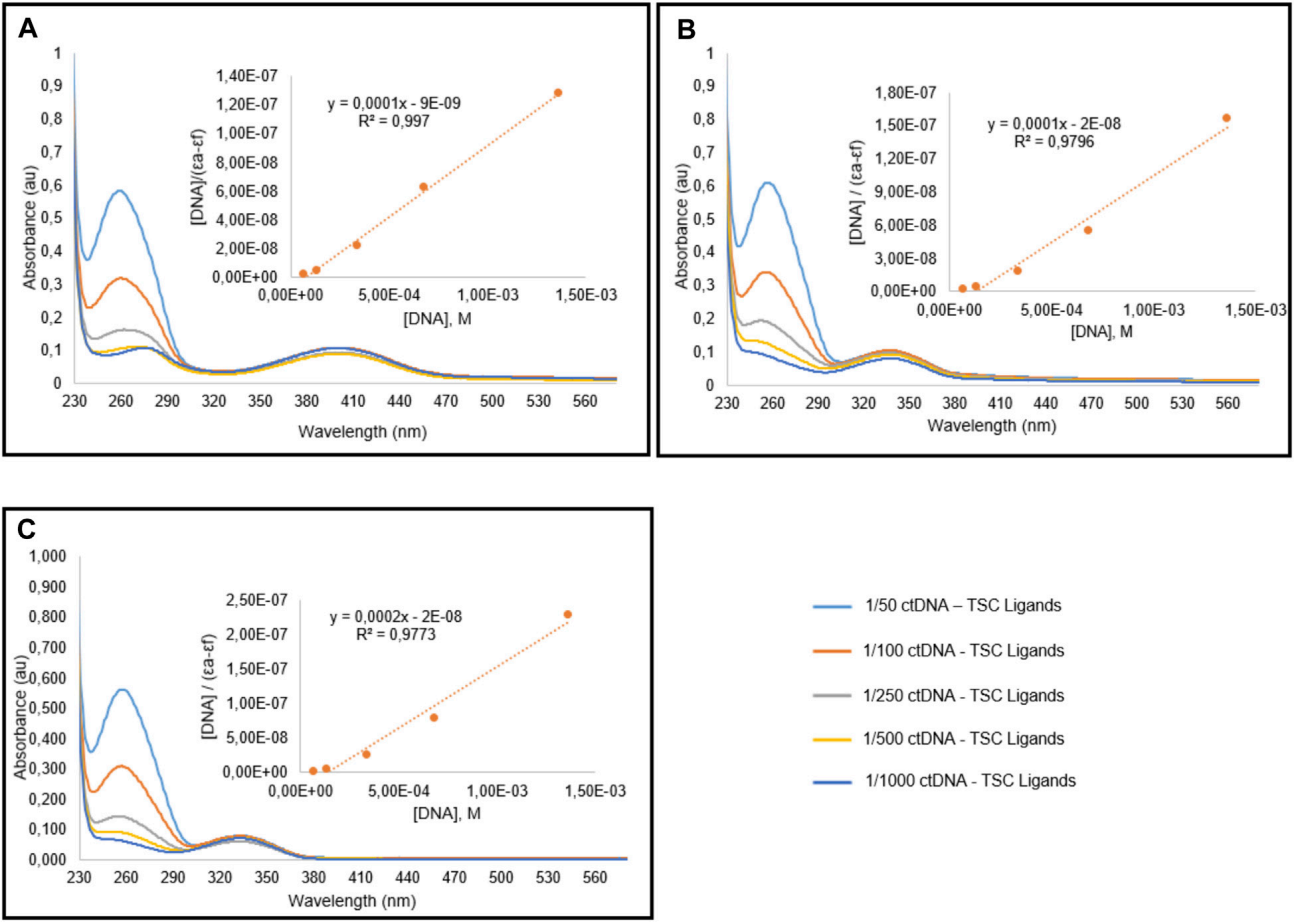
Spectral graphic of TSC ligands L1 **(A)**, L2 **(B)**, L3 **(C)** in the presence of CT-DNA. Ligands concentration is 10 μM, and DNA concentration is variying between 0.07 and 1.4 mM.

**FIGURE 5:**
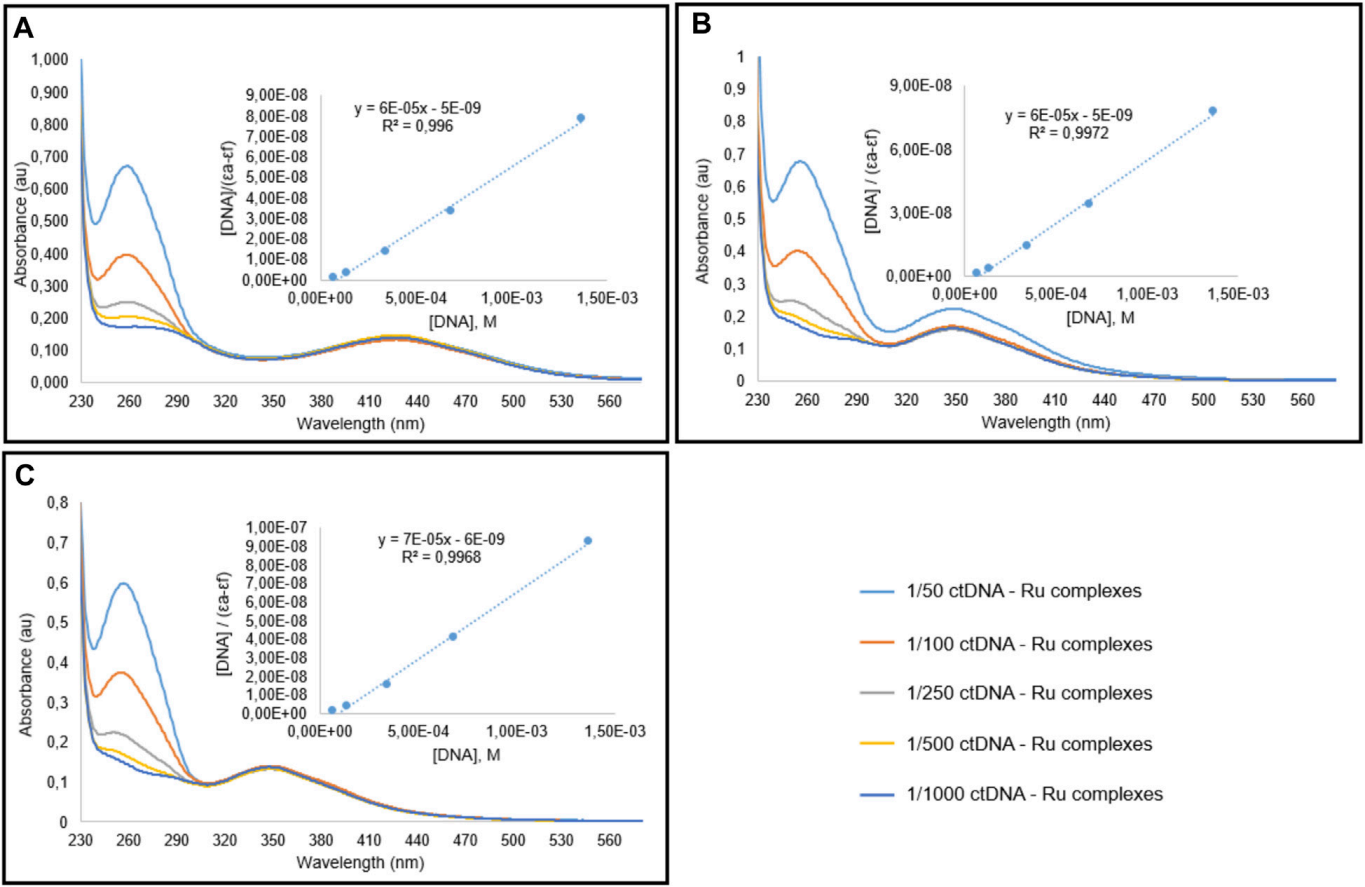
Spectral graphic of Ru complexes I **(A)**, II **(B)**, III **(C)** in the presence of CT-DNA. Complex concentration is 10 μM, and DNA concentration is variying between 0.07 and 1.4 mM.

The absorption spectra of TSC ligands and their corresponding Ru(II) complexes I, II, and III treated with different concentrations of calf thymus DNA (CT DNA) were indicated in Figures 4, 5, respectively. The DNA-binding affinities of TSC ligands followed the order of I > III > II. This situation was found different in Ru(II) complexes; I = II > III. Complex III showed the minimum binding activity to CT DNA. DNA binding affinity results were shown in Table 5. The intrinsic binding constants were determined to be 1.2 × 104, 1.2 × 104, and 1.17 × 104 for complex I, II, and III, respectively. Kb values indicated that ruthenium (II) complexes had more DNA binding characteristics than TSC ligands. Isopropyl groups and methyl groups in p-cymene provide strong DNA binding affinity.

**TABLE 5:** DNA binding constant (K_b_) values for TSC ligands and Ru complexes.

DNA binding	Molecules	K_b_(M^−1^)
Ligands	L1	1.11 × 10^4^
	L2	5 × 10^3^
	L3	1 × 10^4^
Ruthenlum Complexes	I	1.2 × 10^4^
	II	1.2 × 10^4^
	III	1.17 × 10^4^

### 3.5 Protein Binding Results

**FIGURE 6:**
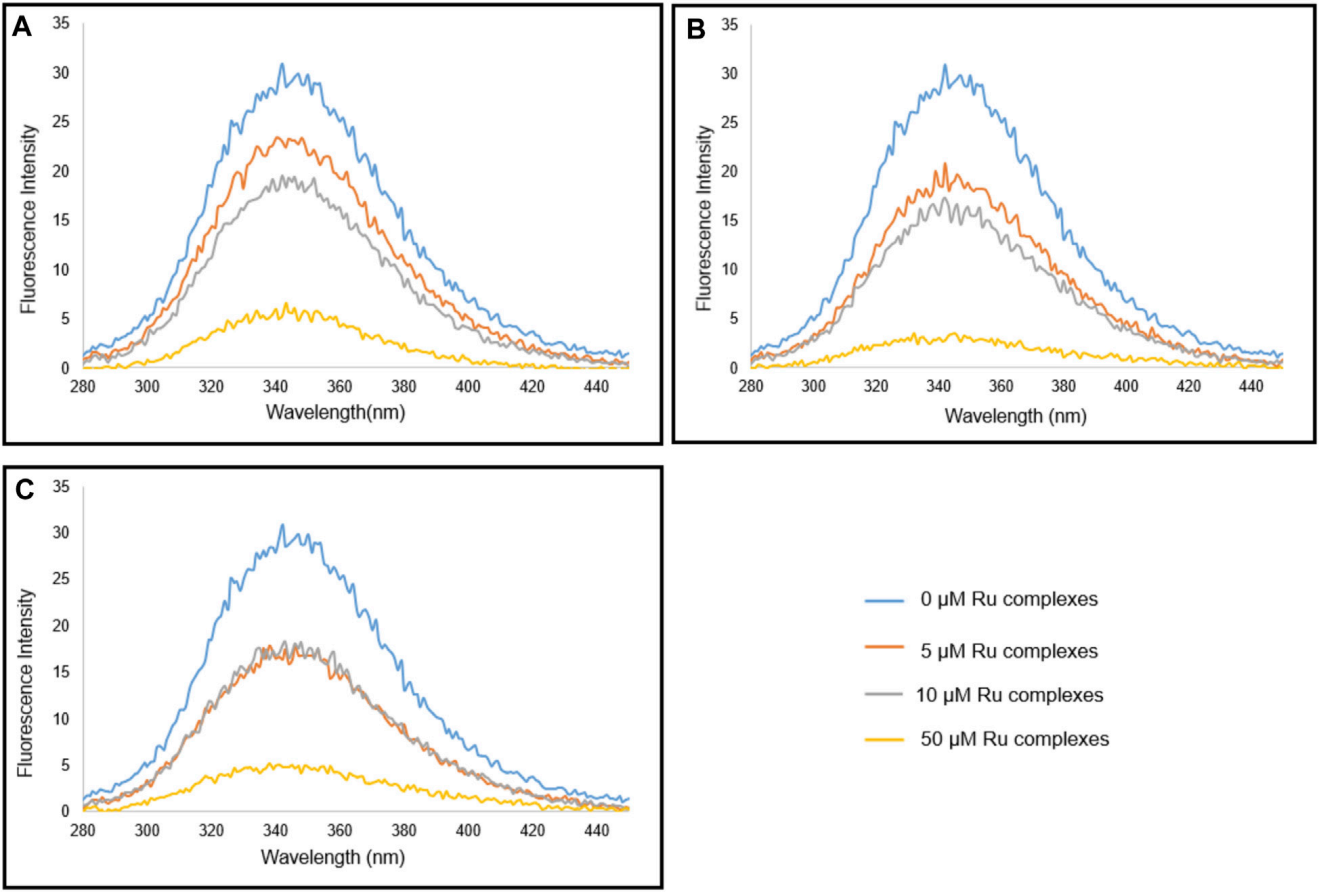
Spectra graphic of 1 μM BSA in Ru complexes, I **(A)**, II **(B)**, III **(C)**.

Serum albumin is one of the main proteins found in plasma. Since it is found easily and its structure is similar to human serum albumin, bovine serum albumin (BSA) is widely used in protein binding studies. BSA binding studies give information about pharmacokinetics and the biological distribution of molecules in the body. The binding of organo ruthenium complexes with BSA was evaluated using fluorescence spectra. The spectral emission was recorded at 280 nm excitation wavelength. The emission was monitored between 250 and 450 nm wavelength. Experiments were carried out by titrating BSA solution (1 μM) in varying Ru(II) complex concentrations (0–50 μM) in phosphate buffer (pH 7.2). Figure 6 showed a spectra graphic of 1 μM BSA in Ru complexes. From the spectra, it can be understood that 50 µM Ru(II) concentration utilized in the study indicated the strongest binding value, and other concentrations (5, 10 µM) were found to be less bound to BSA. The balance between bound and free molecules can be represented by the Scatchard equation (log((I_0_-I)/I) = nlog [Q] + logKb) (Lakowicz and Berndt, 1991; Kathiravan et al., 2009). I_0_ shows the density value of BSA; I is the density of Ru(II) complexes that interact with BSA; [Q] denotes the complex concentration, and n shows the number of the binding site and Kb shows the binding constant. The binding constant Kb was determined by plotting according to log[(I_o_-I)/I] corresponding to log[Q]. As seen in Table 6, The binding constants of Ru(II) complexes to BSA were determined to be 7.09 × 10^5^, 7.67 × 10^5^, and 2.03 × 10^4^ for complex I, II, and III, respectively. The binding affinity of Ru(II) complexes to BSA followed the sequence of II > I > III. Taken together, these results showed that the binding of the Ru(II) complexes with BSA may be due to the presence of static quenching (Thota et al., 2016).

**TABLE 6:** K_b_ and n values after the treatment of Ru Complexes (I, II, and III) with BSA.

BSA binding	Molecules	Kb(M^−1^)	n
Ruthenium Complexes	I	7.09 × 10^5^	1.21
	II	7.67 × 10^5^	1.71
	III	2.03 × 10^4^	0.85

### 3.6 DNA Binding Competition Experiments Results

**FIGURE 7:**
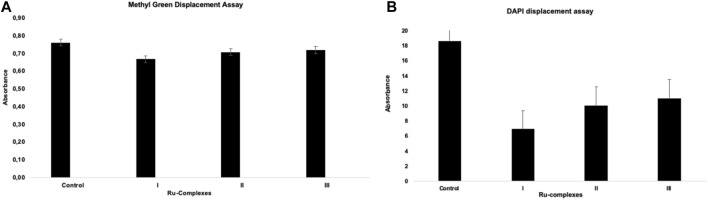
Methyl green **(A)** and DAPI **(B)** absorbance in DNA binding competitive study.

Two different competitive binding studies were studied using DAPI and methyl green to find where the Ru(II) complexes bind on CT-DNA. In both cases, the competition of a second molecule for DNA binding resulted in loss the of absorbance (Pettinari et al., 2014). In these two different competitive binding studies, changes in the DAPI or methyl green absorption in the presence of Ru(II) complexes were evaluated. DAPI is mainly considered the minor groove binder of DNA, while methyl green is the major groove binder of DNA (Kim and Nordén, 1993). Figure 7 showed the absorbances for both methyl green and DAPI displacements. The results indicated that there was no considerable reduction in methyl green-DNA complex absorbance. Taken together, these results may suggesting that Ru(II) complexes may compete with DAPI and these data showed that there was a possibility of binding of Ru(II) complexes to the minor groove of DNA.

### 3.7 Trans-Epithelial Resistance Measurement Results

One of the most important functions of tight junctions between cells is to limit the spread of ions and hydrophilic non-ionic molecules depending on their load and size (Contreras et al., 1992; Chen et al., 2000). Trans-epithelial resistance (TER) measurement is usually studied to determine the ion permeability of these tight junction points (Matter and Balda, 2003). In the recent study, after the cells were placed in transwells with pore diameters of 0.4 μm, they were kept until they reached sufficient confluency. During this period, the TER measurements were taken before changing the medium of the cells every 3 days (No data were shown for the measurement of TER). TER measurements were taken at regular intervals and the values were recorded as numbers. It was observed that the TER values increased day by day. This showed that as the confluency of the cells increased, tight junction points were constructed structurally and functionally. The highest values in TER measurements of OVCAR-3 and A2780 cells taken before applying Ru(II) complexes were 182 and 185Ω, respectively. After applying Ru(II) complexes at, values, TER measurements were taken at regular intervals for 3 days. It was observed that the TER value of OVCAR-3 cells decreased to 140 Ω for complex I. This value was measured as 143 and 145 Ω for complex II and complex III, respectively. In A2780 cells, after applying complex I, the TER value was found 139 Ω, while this value was measured as 165 and 160Ω in complex II and complex III, respectively. These results showed that the transepithelial resistance of cancer cells increased in a time-dependent manner before the administration of Ru(II) complexes. However, after the application of Ru(II) complexes, it was determined that the time-dependent trans epithelial resistance values began to decrease, indicating that ovarian cancer cells, which were administered by the ruthenium (II) complex, started to die and separated from the cell population.

### 3.8 Cellular Uptake Results

**FIGURE 8:**
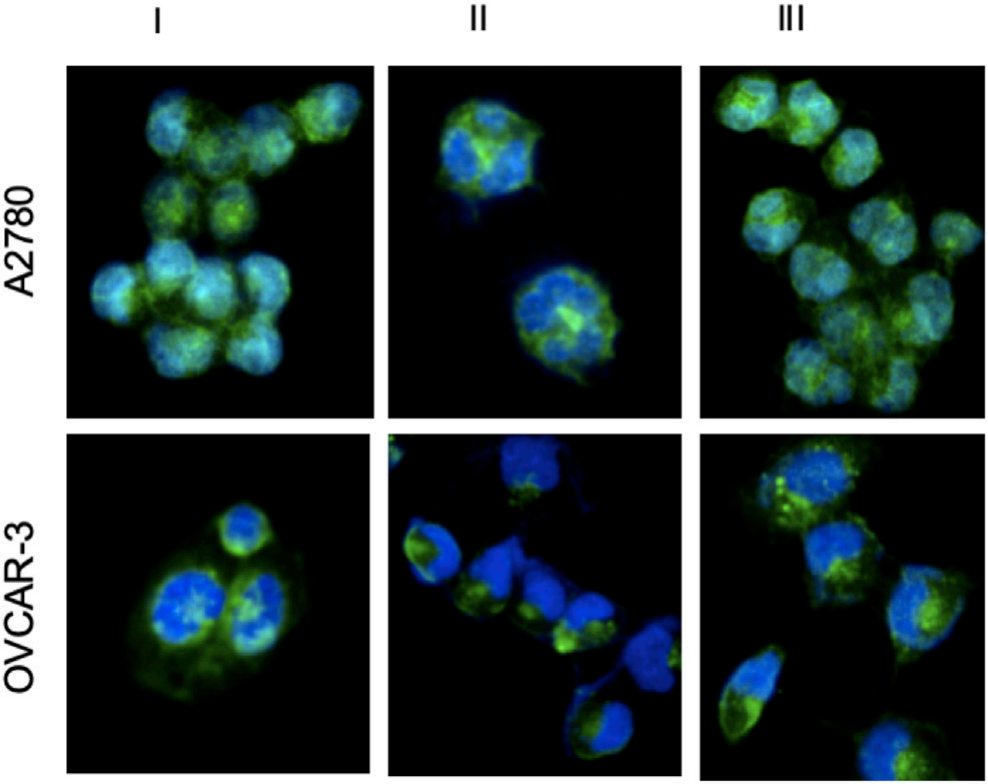
DAPI staining of 24 h after applying Ru complexes to A2780 and OVCAR-3 cells. (Blue for DAPI, Green for Ru complexes) (I, II, III represents for ruthenium complex I, complex II, complex III, respectively).

Cancer cells have more transfer receptors than healthy cells to meet the increased iron need. Since Ru(II) complexes behave similarly to iron metal, they are more effectively taken up by cancer cells (Allardyce et al., 2005; Dyson and Sava, 2006). Therefore, the potential for effective uptake of Ru(II) complexes into the cells should be carefully investigated. A2780 cells and OVCAR-3 cells were incubated with complexes I, II, and III at 20 µM for 24 h 37 C temperature. As shown in Figure 8, the overlay images represented that the blue channel displayed DAPI stained nuclei and the green channel showed the luminescence of complexes I-III. It was demonstrated that the blue channel could fully overlay with the green channel. Taken together, these results strongly suggested that Ru(II) complexes can be uptaken by both cell lines and they penetrated the interior of the nucleus.

## 4 Conclusion

Today, it is known that chemotherapeutic drugs, which are still widely used in cancer treatment, are not fully effective in all types of cancer, they cause drug resistance, and there have many important side effects. These adverse effects have led researchers to focus on metal-based anticancer drugs that show less toxicity and high selectivity to cancer cells. At this point, studies have rapidly increased in the field of metal-based pharmaceuticals in the treatment of cancer cells. Ruthenium complexes, metalopharmaceutical compounds, have shown attractive properties including high ligand exchange rates, higher cytotoxic effects, and high selectivity. Within the scope of this study, TSC ligands (L1, L2, and L3) and arene Ru(II) complexes (I, II, and III) were synthesized and characterized structurally, and their biological potency was also investigated. It was seen that newly synthesized Ru(II) complexes were found to be taken into cancer cells effectively within 24 h, and had a strong cytotoxic effect on ovarian cancer cell lines. Considering the results of this study, it is thought that the newly synthesized organo Ru(II) complexes (I, II, and III) may be potential molecules for effective treatment for the patients who have been diagnosed with ovarian cancer.

## Data Availability

The datasets presented in this study can be found in online repositories. The names of the repository/repositories and accession number(s) can be found below: CCDC ‐ 2015107 (https://www.ccdc.cam.ac.uk/structures/Search?access=referee&ccdc=2015107&Author=Betul+Sen), 2015108 (https://www.ccdc.cam.ac.uk/structures/Search?access=referee&ccdc=2015108&Author=Betul+Sen). Details on how to access this data can be found in the Supplementary Material.
