# Supplementary material for: Half-Sandwich Arene Ruthenium(II) Thiosemicarbazone Complexes: Evaluation of Anticancer Effect on Primary and Metastatic Ovarian Cancer Cell Lines
Source: Front Pharmacol. 2022 May 10;13:882756. doi: 10.3389/fphar.2022.882756 (PMC9128756; doi:10.3389/fphar.2022.882756)
Supplement: Supplementary file 1 [file DataSheet1.pdf]

You can follow the instructions below to get the relevant data for the compounds L1 and L2:

1. Click one of the links provided for L1 or L2.

2. Click "download selected" button

3. From the drop-down list, click on the third option: "deposited file(s) with any available structure factor data and checkCIF reports included"

4. On the same page click "opt-out" option to access the data publicly.

5. On the opened page, click on "i do not want to add user details" button.

6. Accept the terms and conditions

7. Click on download button

This instruction in the given order should work properly.
